# Differential Expression Profile of Chicken Embryo Fibroblast DF-1 Cells Infected with Cell-Adapted Infectious Bursal Disease Virus

**DOI:** 10.1371/journal.pone.0111771

**Published:** 2015-06-08

**Authors:** Raymond K. Hui, Frederick C. Leung

**Affiliations:** 1 School of Biological Sciences, The University of Hong Kong, Hong Kong, People’s Republic of China; 2 Bioinformatics Center, Nanjing Agricultural University, Nanjing, China; George Mason University, UNITED STATES

## Abstract

RNA-Seq was used to unveil the transcriptional profile of DF-1 cells at the early stage of caIBDV infection. Total RNAs were extracted from virus-infected cells at 0, 6 and 12 hpi. RNA-Seq datasets of respective samples mapped to 56.5–57.6% of isoforms in the reference genome Galgal4.73. At 6 hpi, 23 isoforms underwent an elevated expression, while 128 isoforms were up-regulated and 5 were down-regulated at 12 hpi in the virus-infected group. Besides, 10 isoforms were exclusively expressed in the virus-infected cells. Though no significant change was detected in cytokine and interferon expression levels at the first 12 hours of infection, modulations of the upstream regulators were observed. In addition to the reported regulatory factors including EIF2AK2, MX, OAS*A, GBP7 and IFIT, IBDV infection also triggered a IFIT5-IRF1/3-RSAD5 pathway in the DF-1 cells which potentially restricted the viral replication cycle in the early infection stage. Over-expression of LIPA and CH25H, together with the suppression of STARD4, LSS and AACS genes implied a modulation of membrane fluidity and lipid raft arrangement in the infected cells. Alternative splicing of the EFR3 homolog A gene was also through to be involved in the lipid membrane regulation, and these cumulative responses projected an inhibition of viral endocytosis. Recognition of viral RNA genomes and intermediates was presumably enhanced by the elevated levels of IFIH1, DHX58 and TRIM25 genes which possess properties on detecting viral dsRNA. On the other hand, the caIBDV arrested the host's apoptotic process by inducing the expression of apoptosis inhibitors including NFKBIA/Z, TNFAIP2/3 and ITA at the first 12 hours of infection. In conclusion, the differential expression landscape demonstrated with RNA-Seq provides a comprehensive picture on the molecular interactions between host cells and virus at the early stage of infection.

## Introduction

Infectious bursal disease (IBD) has been striking chicken flocks for more than fifty years exerting an considerable economical impact to the global poultry industry. The disease brings a direct mortality ratio up to 90–100% [1, 2], and as it causes destruction of B-lymphocytes in the bursa of Fabricius, it leads into severe immunosuppression and hence secondary infections may result in infected chickens [3, 4, 5]. Infectious bursal disease virus (IBDV) is the causative agent of the disease. Two serotypes are identified in which serotype 1 comprises pathogenic strains, whereas serotype 2 strains cause neither disease nor protection against serotype 1 strains in chickens [6, 7, 8, 9]. It is demonstrated that the virus propagates in the actively proliferating IgM-bearing B-lymphocytes and hence induces apoptotic effects [10, 11, 12]. Though the pathogenicity and epizootiology have been studied for a certain period of time, the molecular interactions between the host cells and the viruses have not been well defined yet. In recent years studies have started to focus on the molecular mechanisms involved in the host responses upon IBDV infection. Quantitative RT-PCR (qRT-PCR) and microarray assays are increasingly employed to reveal the transcriptional changes of the host cells in response to IBDV infections [13–30]. While some studies also utilize proteomic approaches to identify the differentially expressed protein during the course of IBDV infection [31, 32]. Majority of these studies emphasized the cytokine responses including interleukin and interferon expressions, whereas some of these studies revealed expression of mRNA related to apoptotic mechanisms. Up to now, however, there is no comprehensive transcriptional landscape described in the cells upon IBDV infection. In order to explore the differential expression pattern in the event of IBDV infection, RNA sequencing (RNA-Seq) was employed to assay the transcript variations across the entire chicken genome. RNA-Seq reveals a high overall sensitivity on differentially expressed gene level compared with other whole-transcriptome expression quantification platforms including microarrays [33, 34]. The prerequisite of hybridization-based microarray assays relies on existing knowledge about genome sequences [35, 36] and hence limits the detection of novel, rare transcript species exist in the transcriptome. Whereas RNA-Seq takes an advantage not only in determining the differential expression level of transcripts, but it also provides evidence on transcript splice-variants, isoforms and single nucleotide polymorphism (SNPs) [37]. It has also been demonstrated that RNA-Seq is highly accurate for determining gene expression levels as performed with qPCR [38]. Background levels resulting from cross-hybridization is also much lower than occurred in microarray assay [39]. Taking these advantages, in this study we made use of RNA-Seq to unveil the transcriptomic dynamics upon caIBDV infection in DF-1 cells and to reveal a more comprehensive molecular interactions between the host cells and the virus.

## Materials and Methods

### Cell culture and virus

Chicken embryonic fibroblast cells DF-1 (CRL-12203, ATCC) were maintained and cultured with high glucose (4.5g D-Glucose/L) Dulbecco's Modified Eagle Medium DMEM-HG (Life Technologies, NY) supplemented with 10% (v/v) fetal bovine serum at 37°C, 5% CO_2_. Cell-adapted IBDV (caIBDV) was generated with propagating IBDV vaccine strain D78 (VR-2041, ATCC) in secondary chicken embryonic fibroblast cells, followed by purification with CsCl gradient and 20% sucrose gradient as described previously [21]. The quantity of the purified virus was determined by standard plaque assay [40].

### Virus inoculation

DF-1 cells were seeded into each well of 6-well plate (Costar 3516, Corning, NY) at 1 × 10^4^ cells at 24 hrs prior to virus inoculation. A total of six individual wells were prepared for each sampling time point of both mock- and caIBDV- infected groups. The purified caIBDV was diluted to 10 multiplicity of infection (m.o.i.) per ml with DMEM-HG without serum supplement. Before inoculation, culture medium was discarded and the cells were rinsed with 1 × phospate-buffered saline once. One ml of diluted caIBDV was applied into each well of the virus-infected group, whereas DMEM-HG without serum supplement was added in the mock-infected group. At 0, 6, 12 hrs post-infection (hpi), medium was aspirated from the wells of mock- and caIBDV-infected groups. One ml of SV RNA Lysis Buffer (Promega, WI) was added into each well and the cells were dispersed and lysed with repeated pipetting. A total of six individual cell lysates were collected from each treatment group at designated time points and kept at -80°C for total RNA extraction thereafter.

### Total RNA isolation

Total RNAs were extracted from cell lysates with SV96 Total RNA Isolation System (Promega, WI) according to manufacturer's protocol. Total RNAs extracted from each treatment group at the same time point were pooled into one tube for the downstream processes. A total of six RNA samples were obtained representing two treatments at three time points. Quantity of the pooled RNA samples was determined with Quant-iT RiboGreen RNA Reagent and Kit (Molecular Probes, OR), while the quality and integrity of RNA were confirmed with spectrophotometry and formaldehyde 1% agarose gel electrophoresis. All total RNA samples possessed A260/A280 ratio > 1.8 and the 28S:18S rRNA intensity ratio of 1.76 estimated with Nanodrop 2000 (Thermo Scientific, DE) and Quantity One Analysis Software (Bio-Rad, CA) respectively.

### Complementary DNA (cDNA) library construction and normalization

Four micrograms of each pooled total RNA were used for cDNA library construction using TruSeq RNA Sample Prep Kits v2 (Illumina, CA) according to the Low Sample (LS) Protocol of the TruSeq RNA Sample Preparation v2 Guide (Part# 15026495 Rev. D September 2012, Illumina, CA). The libraries were indexed with individual adaptor and normalized with Library Quantification Kit—Illumina/ Universal (Kapa Biosystems, MA) according to manufacturer's instructions. The normalized libraries were then pooled and loaded onto Illumina MiSeq Instrument for cluster generation and sequencing.

### Whole transcriptome shotgun sequencing

A 2 × 250 paired-end sequencing run was conducted using MiSeq Reagent Kit v2, 500 cycles with a standard flow cell (14 tile) (Illumina, CA) on an Illumina MiSeq machine. Raw reads generated from the run were demultiplexed and underwent quality trimming prior to the downstream analytical pipelines. Only reads with Q Score ≥ 30, and aligned with corresponding paired reads were retained for the differential expression analysis.

### Sequence analysis

The paired-end sequence reads of individual sample were aligned and mapped to a chicken reference genome (Gallus Gallus reference genome Galgal4.73) with STAR RNA-Seq aligner 2.3.0 [41] in default setups. Both the unmasked reference genome and the annotations of both coding and non-coding genes were obtained from Ensembl database [42]. The mapped reads were then converted to binary BAM files with SAMtools 1.0.2 [43] for transcript expression levels comparison. The aligned, mapped reads from the same time point of both mock- and caIBDV-infected group were compared and the expression levels at the isoform and gene level were calculated with Cuffdiff package in Cufflink transcript assembly tool [44]. The same annotation and reference genome (Galgal4.73) files as for STAR aligner were used in Cuffdiff pipeline. Expression level of each transcript in the samples were expressed in fragments per kilobase of transcript per million mapped fragments, FPKM. Each observed alternation in transcript expression with p<0.05 was regarded as statistical significant. Quality of the libraries and sequencing performance were examined with RNASeQC tool [45]. The differentially expressed genes were grouped with hierarchical clustering algorithms [46] in the RNA-Seq analysis package of Gene Pattern web-based tool (Broad Institute, MIT).

The potential protein-protein inactions among the genes in the altered expression profiles derived from RNA-Seq analyses were predicted with STRING (Search Tool for the Retrieval of Interacting Genes/Proteins) v9.05 [47].

In addition to the chicken reference genome, the paired-end sequence reads of each sample group were also mapped to a IBDV reference sequence (IBDV Segment A, NC_004178.1; IBDV Segment B, NC_004179.1 [48]) with GS Reference Mapper in 454 Sequencing System Software Package v2.7 (Roche, CT) in order to assess the change of caIBDV viral load in the infected DF-1 cells.

### Nucleotide sequence accession number

The RNA-Seq datasets have been deposited at NCBI Gene Expression Omnibus (GEO) repository under the accession number of GSE60268.

## Results and Discussion

### Infection experiment and RNA-Seq

To study the transcriptomic profile of chicken embryonic fibroblast DF-1 cells during caIBDV infection, caIBDV strain D78 at 10 m.o.i. was used to infect DF-1 cells for 0, 6 and 12 hours. The relatively high dosage of virus was used for inoculation as to minimize the influence exerted from a large number of uninfected cells [49].

Total RNAs were isolated from both mock- and caIBDV-infected groups at respective time points and cDNA libraries were constructed for a 2 × 250 paired-end sequencing run on an Illumina MiSeq machine. The sequence run yielded a total of about 32 millions passed-filter reads, and 85.1% of the reads possessed Q-Score ≥ 30. In average, > 4 million quality-trimmed reads were generated for each sample (Table 1). Though it has been suggested that high-quality eukaryotic transcriptome reconstruction requires more than ten million reads for discovering new genes and transcripts [34], the relatively shallow RNA-Seq datasets are otherwise sufficient to provide accurate differential expression trends of transcript as confirmed with qRT-PCR [50, 51, 52]. Hence RNA-Seq datasets obtained in this study can illustrate the evidence on differential expression landscape between the mock- and virus-infected conditions.

**Table 1:** Statistics on the RNA-Seq datasets.

		Mock-infected			caIBDV-infected	
hpi		Raw read	Trimmed read		Raw read	Trimmed read
0	R1	2,697,493	2,274,144	R1	2,548,186	2,140,585
	R2	2,953,303	2,500,845	R2	2,802,826	2,368,373
6	R1	2,643,354	2,250,936	R1	2,524,045	2,166,168
	R2	2,861,666	2,445,301	R2	2,730,637	2,351,470
12	R1	2,681,507	2,257,774	R1	2,334,929	2,004,679
	R2	2,939,553	2,488,528	R2	2,547,933	2,196,213

### Viral replication dynamics

**Fig 1:**
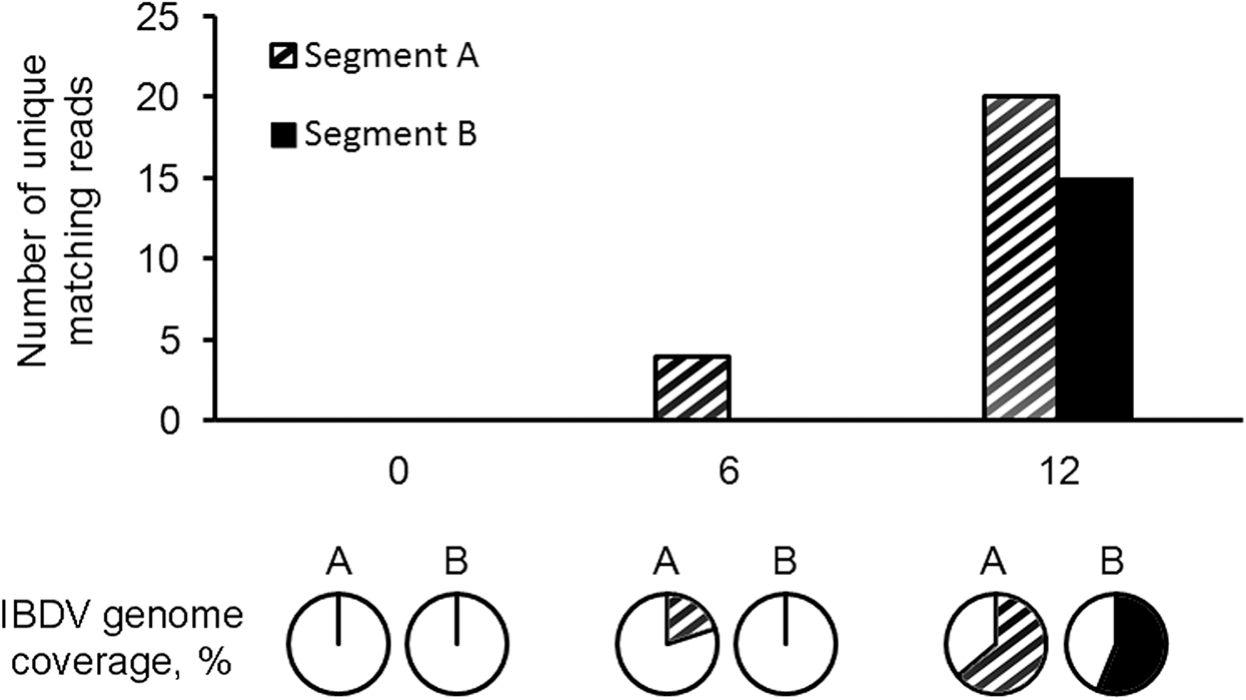
Number of unqiue matching reads and percentage of genome coverage to IBDV reference genome. The demultiplexed, trimmed sequence reads were mapped to IBDV reference genome comprising two segments. No IBDV sequences were identified in the mock-infected group and at 0hpi of the caIBDV-infected group. The sequence reads mapped to segment A showed up at 6hpi, with 20.01% coverage of the segment. While at 12hpi, both segment A and B sequences were mapped covering 63.87% and 56.17% of the respective genome segments. Shaded: segment A; solid: segment B.

After demulitplexing and quality trimming, the six datasets were mapped to IBDV reference genome (Segment A, NC_004178.1; IBDV Segment B, NC_004179.1) with GS Reference Mapper Algorithms (Roche, CT). The mapping matrix indicates that no sequence read from the mock-infected group matched to the reference genome. On the other hand, unique sequence reads of the segment A of the IBDV genome was detected in 6hpi and 12hpi, whereas those matched to segment B was observed at 12hpi (Fig 1). The sequence reads covered 20.01% to 63.87% of the entire segment A sequence at 6hpi and 12hpi samples respectively, while 56.17% of segment B sequence was retrieved from the 12hpi sample. This finding reveals that the replication machinery of caIBDV started as early as at 6 hours after the inoculation. The segment A sequences identified in the dataset did not only indicate the replication of IBDV genomic RNAs, but it also reflected the transcription of sub-genomic RNA species encoding for the viral structural proteins VP2, 3, 4 and 5 for the assembly of a mature virion. While the segment B sequences were observed at 12hpi, indicating that there was an expression of viral RNA polymerase VP1 and also the replication of this segment. The increase of mapped sequence reads indicates an active viral replication process in this 12-hours post inoculation period.

### Differential expression patterns

**Fig 2:**
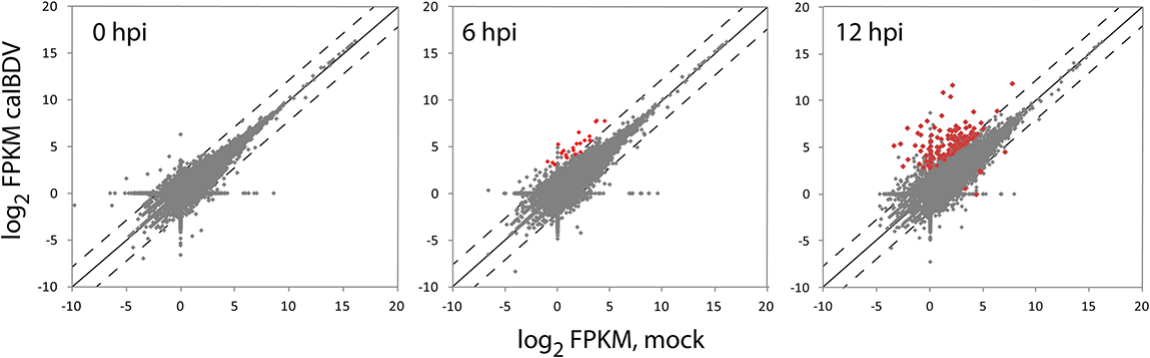
Pair-wise comparison of transcript expression in mock- and caIBDV-infected DF-1 cells at 0, 6 and 12 hours post-infection. Comparison of expression data was performed by XY-scatterplot analysis of log base 2-transformed value of fragments per kilobase of transcript per million mapped fragments, FPKM. The solid line represents the predicted line of identity. The dashed line indicates the threshold of ≥ 2-fold or less than one-half of expression ratios. Data points shown in red represents significant differentially expressed transcripts, p < 0.05.

**Fig 3:**
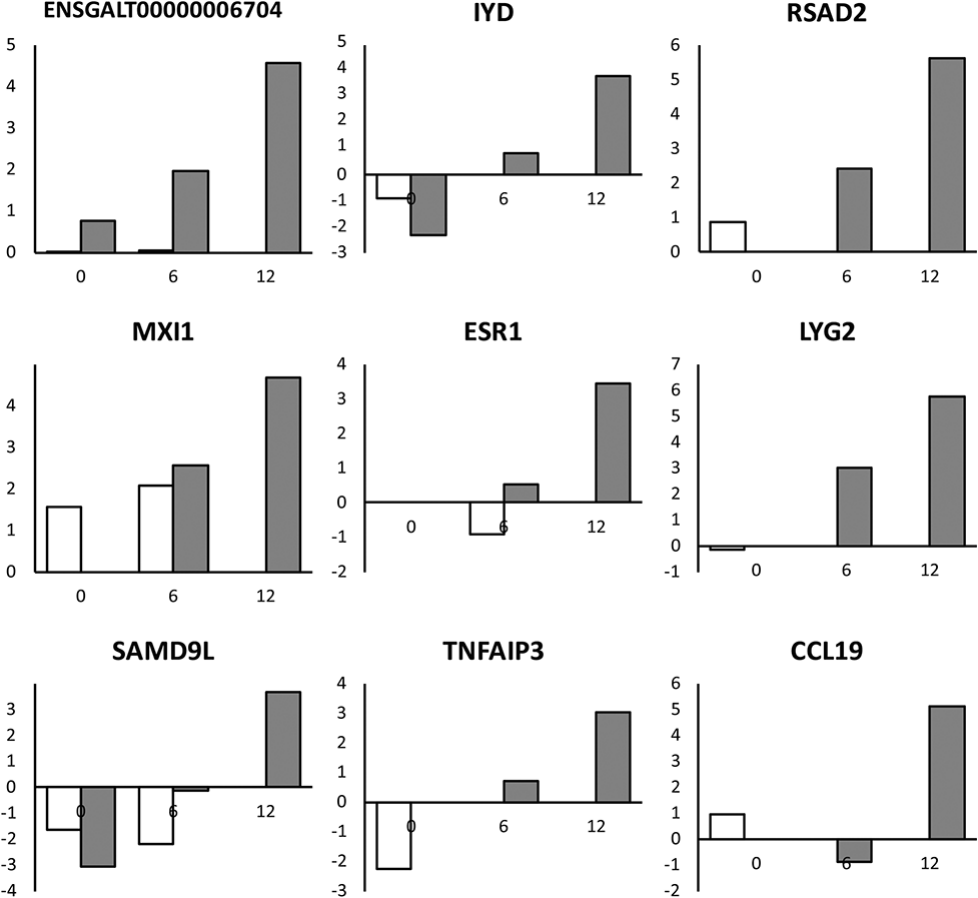
Nine transcripts expressed in caIBDV-infected cells only at 12 hpi. The expression level of these nine transcripts increased gradually from 0 to 12 hpi. ENSGALT00000006704: torsin family 1, member B-like mRNA; IYD: iodotyrosine deiodinase; RSAD2: radical SAM domain-containing 2, viperin; MXI1: MAX-interacting protein 1; ESR1: estrogen receptor 1; LYG2: lysozyme G-like 2; SAMD9L: sterile alpha motif domain containing 9-like uncharacterized protein; TNFAIP3: Tumor necrosis factor, alpha-induced protein 3; CCL19: Chemokine (C-C motif) ligand 19. White: mock-infected group; grey: caIBDV-infected group. x-axis: hours post-inoculation; y-axis: transcript level expressed as log2(fold change).

**Fig 4:**
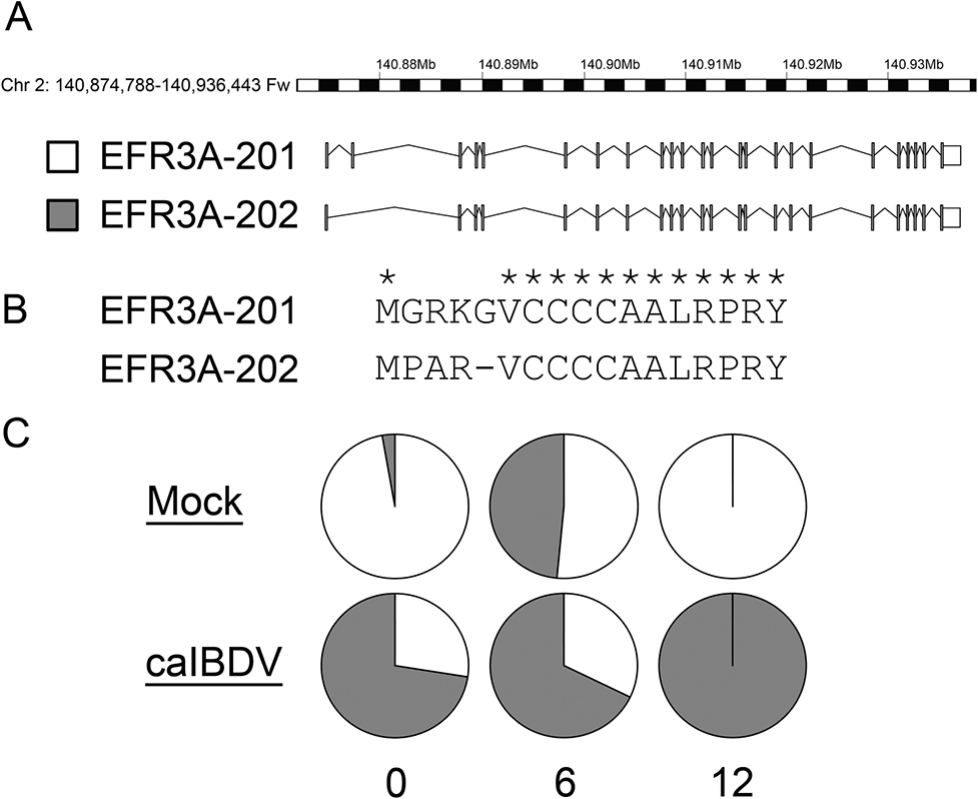
Switching expressions of alternatively spliced froms of EFR3A gene. (A) Structure and chromosomal location of the two transcripts of EFR3A gene; (B) Amino acid residue alignment of the N-terminal of the two transcripts; (C) Expression level (% of FPKM value) of the two transcripts in mock- and caIBDV-infected groups. White: EFR3A-201; grey: EFR3A-202.

To identify the differentially expressed transcripts in DF-1 cells upon caIBDV infection, the six RNA-Seq datasets were then aligned and mapped to a chicken reference genome Ensembl Galgal4.73 with STAR-aligner. The numbers of genes and isoforms mapped to the reference genome respective to each dataset remained constant throughout the course of experiment (Table 2). The datasets mapped to 57.7–58.9% of genes and 56.5–57.6% of isoforms in the reference genome. There was no significant alternations in the expressed gene/ transcript isoform number at the early infection stage (0–12 hpi) of caIBDV infection. Expression levels of the detected transcript isoform from mock- and caIBDV-infected groups were compared with Cuffdiff pipeline. The scatterplot in Fig 2 describes the expression profiles of global differential expressed transcripts at these time points. It was demonstrated that there was no statistical significant difference between the transcriptional profiles of these two groups at 0 hpi, whereas a total of 23 isoforms (0.12%, 23/17,942) were significantly up-regulated at 2.22–5.19 fold (p < 0.05) in the caIBDV-infected group at 6 hpi. The expression levels of 128 transcripts in the virus-infected group were significantly altered (123 up-regulated from 2.07 to 9.7487 fold; 5 down-regulated from 2.06 to 2.61 fold, p < 0.05) compared to mock-infected group at 12 hpi. Besides, 9 additional isoforms were detected in the caIBDV-infected cells only (Fig 3). The result also demonstrates that there was a switch of alternatively spliced transcripts of EFR3A gene between mock- and caIBDV-infected groups (Fig 4).

**Table 2:** Number of genes and transcript isoforms mapped to reference genome Galgal4.73.

	Mock-infected			caIBDV-infected		
	0 hpi	6 hpi	12 hpi	0 hpi	6 hpi	12 hpi
Gene	10,081	9,960	9,980	9,879	9,920	9,907
Isoform	10,350	10,228	10,245	10,147	10,194	10,181

**Fig 5:**
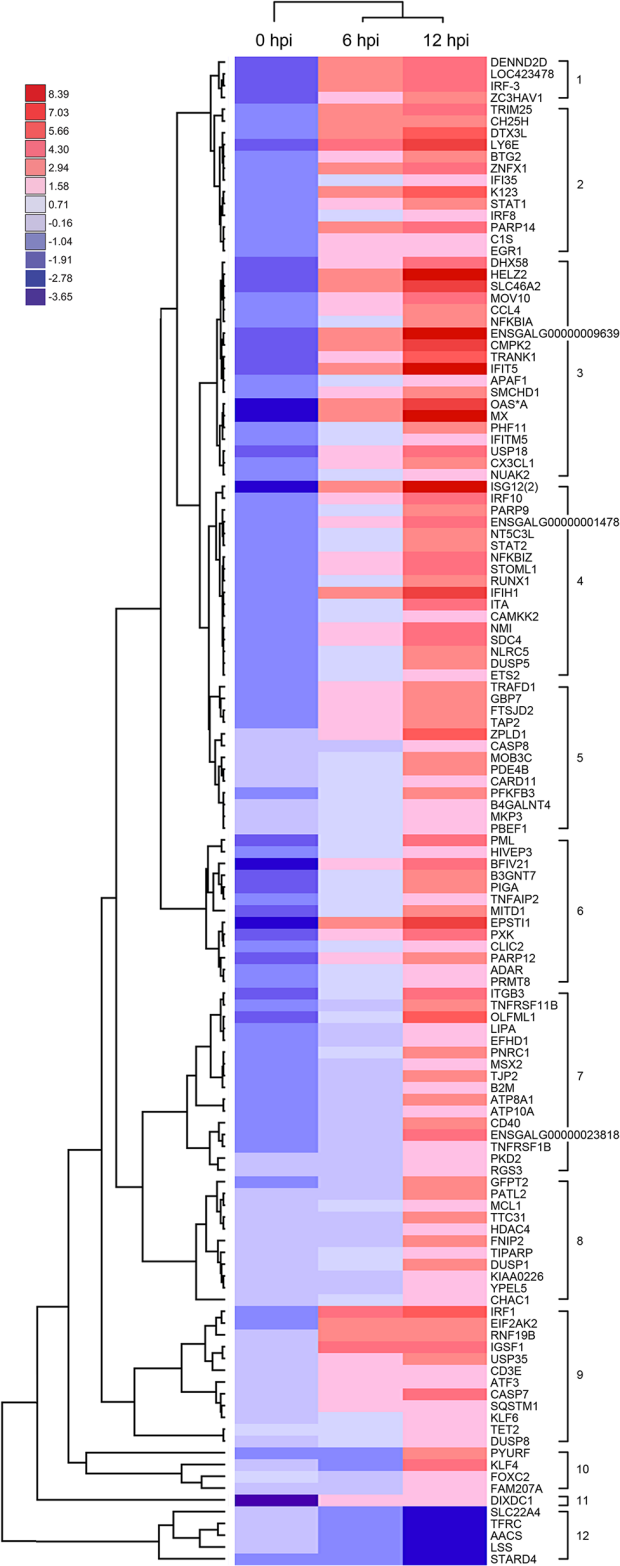
Heatmap indicating the pattern of 128 significant differentially expressed transcripts at 0, 6 and 12 hpi time points. Expression fold change (log_2_ caIBDV/mock) are represented as indicated in the color scale. The transcripts were classified into 12 clusters according to their expression patterns.

The differentially expressed isoforms were classified into 12 clusters according to their expression patterns in hierarchical clustering (Fig 5). Early expressed transcripts (i.e., at 6 hpi) were observed in mainly in cluster 1, 2, 3 and 9, while cluster 12 comprised 5 down-regulated isoforms. Despite most of the expression patterns of these clusters are similar to each other and show limited co-relation with the potential biological process activated by the caIBDV-infection event according to their GO term accessions (Table 3), the result reveals that transcripts involved in anti-viral responses, transcription regulation and membrane proteins were dominantly expressed in caIBDV-infected cells at the very early stage of infection. While transcripts related to apoptotic activities, interferon-dependent immune responses and inflammatory responses were tuned up at 12 hpi.

**Table 3:** Differentially expressed transcripts and the respective biological process upon caIBDV infection in DF-1 cells. Transcripts with fold change at 12 hpi ≥ 4.00 are listed.

				Fold Change		
Ensembl transcript ID	Symbol	Description	Biological function	6hpi	12hpi	Cluster
ENSGALT00000010311	IFIT5	Interferon-induced protein with tetratricopeptide repeats 5	Anti-viral response	4.05	9.75	3
ENSGALT00000022096	ISG12(2)	Putative ISG12-2 protein	Anti-viral response	3.24	9.65	4
ENSGALT00000025999	MX	Interferon-induced GTP-binding protein Mx	Anti-viral response	3.93	9.30	3
ENSGALT00000015699	ENSGALG00000009639	Uncharacterized protein	Anti-viral response	-	8.69	3
ENSGALT00000009906	HELZ2	Helicase with zinc finger 2, transcriptional coactivator	Transcription regulation	4.03	8.60	3
ENSGALT00000018067	IFIH1	Interferon-induced helicase C domain-containing protein 1	Anti-viral response	3.01	8.27	4
ENSGALT00000043751	CMPK2	Cytidine monophosphate (UMP-CMP) kinase 2, mitochondrial	Anti-viral response	3.34	8.26	3
ENSGALT00000022305	OAS*A	59 kDa 2'-5'-oligoadenylate synthase-like protein	Anti-viral response	3.49	8.20	3
ENSGALT00000027416	EPSTI1	Epithelial stromal interaction 1	Apoptosis	-	7.76	6
ENSGALT00000026025	LY6E	Lymphocyte antigen 6E precursor	Transcription regulation	4.50	7.40	2
ENSGALT00000043789	SLC46A2	Solute carrier family 46, member 2	Undescribed	-	7.31	3
ENSGALT00000019695	TRANK1	Tetratricopeptide repeat and ankyrin repeat containing 1	Membrane protein/ receptor	2.90	6.83	3
ENSGALT00000019727	DTX3L	Deltex 3-like protein	Transporter	3.76	6.71	2
ENSGALT00000002244	IRF1	Interferon regulatory factor 1	Undescribed	5.19	6.29	9
ENSGALT00000010244	K123	K123 protein precursor	Respnose to DNA damage	4.00	6.00	2
ENSGALT00000024768	ZPLD1	Zona pellucida-like domain containing 1	Anti-viral response	-	5.91	5
ENSGALT00000044367	OLFML1	Olfactomedin-like 1	Undescribed	-	5.88	7
ENSGALT00000000519	ITGB3	Integrin beta-3 precursor	Undescribed	-	5.62	7
ENSGALT00000001439	IGSF1	Immunoglobulin superfamily member 1	Undescribed	4.36	5.60	9
ENSGALT00000002240	ENSGALG00000001478	Uncharacterized protein	Membrane protein/ receptor	-	5.48	4
ENSGALT00000019721	PARP14	Poly (ADP-ribose) polymerase family, member 14	Membrane protein/ receptor	3.19	5.13	2
ENSGALT00000000210	DENND2D	DENN/MADD domain containing 2D	Undescribed	-	5.04	1
ENSGALT00000007763	ZNFX1	Zinc finger, NFX1-type containing 1	Transcription regulation	3.08	5.00	2
ENSGALT00000021310	USP18	Ubiquitin carboxyl-terminal hydrolase 18	GDP-GTP conversion	2.57	4.96	3
ENSGALT00000005325	DHX58	DEXH (Asp-Glu-X-His) box polypeptide 58	Undescribed	-	4.93	3
ENSGALT00000006265	SDC4	Syndecan-4 precrusor	Interfereon Induced	-	4.82	4
ENSGALT00000018671	LOC423478	Exocyst complex component 3-like	Anti-viral response	3.39	4.77	1
ENSGALT00000002226	STOML1	Stomatin (EPB72)-like 1	B-cell	-	4.74	4
ENSGALT00000044107	BFIV21	MHC BF2 class I precursor	Cellular balance	-	4.69	6
ENSGALT00000002368	MOV10	Putative helicase MOV-10	Undescribed	2.22	4.68	3
ENSGALT00000042652	KLF4	Kruppel-like factor 4	Peptide antigen binding	-	4.62	10
ENSGALT00000011423	PXK	PX domain containing serine/threonine kinase	Antiviral response	-	4.55	6
ENSGALT00000039921	HSPB1	Heat shock protein 25	RNA-mediated gene silencing	-	4.55	7
ENSGALT00000002125	PML	Promyelocytic leukemia	Transcription regulation	-	4.53	6
ENSGALT00000039023	IRF-3	Interferon regulatory factor 3	Apoptotic activity	3.05	4.49	1
ENSGALT00000024766	NFKBIZ	NF-kappa-B inhibitor zeta	Inflammatory responses	-	4.49	4
ENSGALT00000004971	TRIM25	Tripartite motif containing 25	Stress resistance	3.32	4.49	2
ENSGALT00000036426	ITA	Inhibitor of apoptosis protein	Transcription regulation	-	4.47	4
ENSGALT00000020390	NMI	N-myc (and STAT) interactor	Respnose to DNA damage	-	4.35	4
ENSGALT00000014519	CASP7	Caspase 7, apoptosis-related cysteine peptidase		-	4.35	9
ENSGALT00000010406	IRF10	Interferon regulatory factor 10	Interfereon-dependent immune responses	-	4.32	4
ENSGALT00000019729	PARP9	Poly (ADP-ribose) polymerase family, member 9	NF-kappa-B cascade	-	4.21	4
ENSGALT00000040649	CCL4	Chemokine-like ligand 1 precursor	Apoptotic activity	-	4.19	3
ENSGALT00000010225	PFKFB3	6-phosphofructo-2-kinase/fructose-2,6-biphosphatase 3	Inflammatory responses	-	4.13	5
ENSGALT00000001016	GBP7	Guanylate binding protein 7	Anti-viral response	-	4.10	5
ENSGALT00000022552	ZC3HAV1	Zinc finger CCCH-type antiviral protein 1	Apoptotic activity	2.51	4.04	1

### Anti-viral responses exerted by DF-1 cells

**Fig 6:**
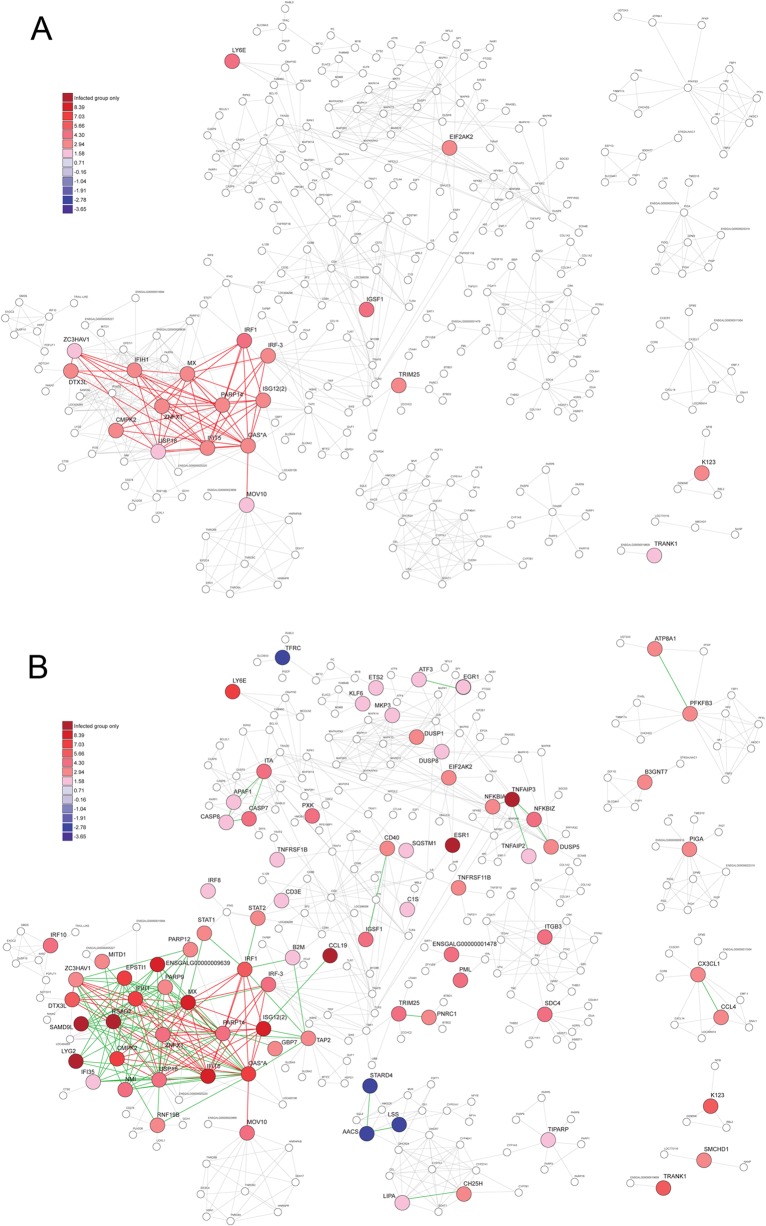
Gene pathways analysis. The potential interaction among the significant differentially expressed isoforms (p < 0.05) were analysed with STRING tool v9.05. (A) 6 hpi; (B) 12 hpi. Expression fold change (log_2_ caIBDV/mock) are represented as indicated in the color scale. Red lines: potential protein interactions started at 6 hpi; green lines: potential protein interactions started at 12 hpi.

It has been well demonstrated that host cells execute a cascade of machinery to combat against viral particle invasion. Previous studies have shown that IBDV infection triggered expression of cytokine genes that are typically up-regulated in general viral infections. Expression of T-helper 1 cell (Th1) cytokine genes including interferon gamma (IFN-γ), interleukin-2 (IL-2), interleukin-12 (IL-12), signal transducers and activators of transcription 1 (STAT1) and 4 (STAT4) were found increased in IBDV-infected chicken or chicken embryonic fibroblast cells at 1–7 days post infection [16, 19, 20, 22, 23, 24, 27, 30]. While elevation of Th2 cytokine genes expressions including IL-4, IL-5, IL-13 was also reported [24]. Genes of cytokine that initiate inflammatory responses including IL-8, nitric oxide synthase (iNOS), and cyclooxygnease (COX-2) were also shown to be up-regulated [17, 18, 27]. In this study, however, no significant elevation of expression levels of these cytokine genes was identified in caIBDV-infected cells. In contrary, the regulatory factors modulating these cytokines including the well characterized eukaryotic translation initiation factor 2-alpha kinase 2 (EIF2AK2, or protein kinase R PKR), interferon-induced GTP-binding protein Mx1 (MX), 59 kDa 2'-5'-oligoadenylate synthetase-like protein (OAS*A), guanylate binding protein (GBP7) and interferon-induced protein with tetratricopeptide (IFIT) were profoundly expressed. Among the 139 differentially expressed transcripts, IFIT5 (interferon-induced protein with tetratricopeptide repeats 5) was expressed in the highest fold change during caIBDV infection. IFIT5 is a member of IFIT1 family which expression can be induced by virus infection, interferons, dsRNA and lipopolysaccharides [53, 54]. It was demonstrated that IFIT5 potentiates anti-viral responses by promoting the interferon regulatory factor 3 (IRF3)- or nuclear factor kappa-light-chain-enhancer of activated B cells (NF-κB)-mediated gene expressions [55]. Over-expression of IFIT5 in the infected DF-1 cells was observed accompany with the elevation of IRF3 at 6 hpi, and progressively with other IRF-3-mediated genes at 12 hpi (Fig 6). One of the dominant anti-viral components potentially triggered by ITIF5-IRF3 pathway was RSAD2 (radical SAM domain-containing 2, viperin) gene. RSAD2 gene was found expressed only in caIBDV-infected cells but not in mock-infected cells at 12 hpi (Fig 3). It is an endoplasmic reticulum-associated virus inhibitory protein which can be induced by type I, II and III IFNs, double-stranded (ds) DNA, dsRNA analog poly I:C, lipopolysaccharide (LPS) and infection of various viruses [56–61]. Besides, it was shown that RSAD2 could be induced via an IFN-independent pathway by transcription factor IRF-1 [62]. Definite anti-viral machineries exerted by RSAD2 has not been concluded yet, but it is believed that it involves in inhibiting viral replication indirectly by altering the cell survival control [63], and membrane fluidity modulation which prevents the budding of viruses [64]. So the switching-on of RSAD2, presumably by ITIF5-IRF3 or by IRF1 regulation, may contribute to the early viral defense in IBDV infection.

Apart from the defense effectors, genes possessed with accessory anti-viral functions were also regulated in caIBDV-infected cells. The interferon-induced helicase C domain-containing protein 1 (IFIH1, or melanoma differentiation-associated protein 5, MDA5) was found expressed in 3.01 and 8.27 fold at 6 and 12 hpi respectively. It was reported that IFIH1 interacts with probable ATP-dependent RNA helicase (DHX58, or Laboratory of Genetics and Physiology 2, LGP2; expressed to 4.93 fold at 12 hpi, Table 3) as a pattern recognition receptor to sense viral dsRNA and triggers downstream antiviral reactions [65, 66]. In addition, elevated expression of tripartite motif-containing protein 25 (TRIM25) at the very beginning of the infection event was believed to be related to the interaction with retinoic acid-inducible gene 1 (RIG-I) in detecting the viral RNA intermediates [67, 68]. Apart from the regulation of RSAD2, the host cells membrane condition during caIBDV infection was probably influenced with cellular cholesterol metabolism. An over-expression of lipase A (LIPA) and cholesterol 25-hydroxylase (CH25H), together with the down-regulation of StAR-related lipid transfer protein 4 (STARD4), lanosterol synthase (LSS) and acetoacetyl-coA synthease (AACS) (Fig 6B), imply an evidence on the modulation of intracellular cholesterol contents in the infected cells. The synergetic effect of the regulation of these genes may alter the lipid raft arrangement and therefore inhibit membrane fusion event between the infected cells and virus, limit the clathrin- and cholesterol-dependent endocytosis hence prevent the propagation of the virus [69, 70, 71].

### Potential strategies used by caIBDV to outwit host cell defenses

Viruses make use of various mechanisms to escape from the host defense actions. One of the viral strategies is to arrest the apoptosis initiation so as to facilitate viral replication process. Apoptosis is one of the host defense mechanism to minimize the spread of viruses. Our previous study demonstrated that apoptosis occurred at 48 hpi [21], while Jungmann *et al*. revealed that the IBDV-induced apoptosis was first observed at 12 hpi [72]. It has been well studied that NF-κB mediated apoptotic pathway is initiated upon viral infection. In uninfected cells, the dimer of NF-κB are sequestered in the cytoplasm by a family of κB inhibitors (IκB). Upon virus infection, these IκB proteins undergo signal-induced degradation by proteasome triggered by the activation of IκB kinase (IKK) and the NF-κB complex is released into the nucleus for the expression of specific genes leading into apoptosis and other antiviral functions. In this study, expression of both NF-κB1 and NF-κB2 were observed in mock- and caIBDV-infected DF-1 cells, but there was no significant changes between both groups at the time points tested (though the trends of both NF-κB proteins increased with time in caIBDV-infected group). Besides, the expression of two IKKs (IKK1 or CHUK and IKK2 or IKBKB) also showed no significant differences, implying that the degradation capacity of IκB between two groups were similar. The result, however, demonstrated that there was a distinct elevation of two IκB levels (NFKBIA or IκBα and NFKBIZ or IκBz) at 12 hpi in the caIBDV-infected group. The increase of these IκBs potentially hindered the function of IKKs and eventually reduced the free NF-κB amount, hence retarded the apoptotic process. Apart from the IκBs, the levels of tumor necrosis factor, alpha-induced protein 2 (TNFAI2) and 3 (TNFAIP3) were also provoked in the infected cells. It has been demonstrated that TNFAIP3 is a potent cellular inhibitor of NF-κB activation [73, 74]. Furthermore, the caspases-mediated apoptosis was probably inhibited by the over-expressed baculoviral IAP repeat-containing protein 2 (BIRC2 or ITA) in the infected cells. ITA is a member of the inhibitor of apoptosis family that inhibit apoptosis by interfering with the activation of caspases [75, 76]. Liu *et al*. suggested that the nonstructural protein (NS) of IBDV possessed anti-apoptotic function at the early stage of virus infection [77]. It is therefore reflecting that the NS of caIBDV regulates the expression of IκB, TNFAIPs and ITA in the infected cells which aims to delay the apoptosis in the first 12 hpi and reserve viable host cells for viral replication.

Switching of alternatively spliced EFR3A transcript isoforms

RNA-Seq data reveals that two splice variants of EFR3 homolog A (*S*. *cerevisiae*) (EFR3A) transcript were identified in mock- and caIBDV-infected DF-1 cells respectively (Fig 4). It was shown that EFR3A-201 expressed in mock-infected cells, while the expression of EFR3A-202 isoform took over the previous one at 12 hpi in the caIBDV-infected cells. Amino acid alignment reveals that four residue differences located at the N-terminal immediately downstream to the first methionine residue between the two variants. EFR3A gene encodes a membrane protein [78], but the definite cellular function of EFR3A in chicken cells has not been reported yet. Odrowaz et al. reported EFR3A participated in the negative control of a ETS transcription factor ELK1 in a human epithelial cell MCF-10A [79]. Whereas it was shown to be involved to the epidermal growth factor receptor signaling pathway [80]. It was also suggested that the differential expression of EFR3A gene in auditory brainstem neurons of mice with hearing deficit [81]. The switch of splice variant expression may be involved in altering the lipid membrane condition, but the molecular mechanism of this deviations to host-virus interaction needs further characterization.

## Conclusion

Interactions between IBDV and its host has been extensively studied. A number of researches revealed that infection of chicken cells, including fibroblast and bursal cells, with IBDV may lead into an elevation of cytokines and interferons at 1 dpi to 7 dpi periods in general. Virus-induced apoptosis via caspase- and NF-κB-mediated pathways were also demonstrated. This study, on the other hand, disclosed the early host-virus interactions. With the aid of RNA-Seq, a more comprehensive expression landscape was obtained. The result presents the events occurred before the elevation of downstream effectors. Apart from the regulators of cytokines and interferons, modulations were observed in the gene candidates involved in cell membrane fluidity. It is believed that the changes in membrane conditions contributes to the frontline host response against endocytosis of IBDV and hence prevent infections. On the other hand, the intensively expressed anti-apoptotic genes induced by caIBDV delay the programmed cell death and hence prolong the viral replication cycle in the host cells.

This study explore the initial host response in DF-1 cells upon caIBDV infection and the potential virus strategy to counteract with the host's action.
